# “Tensions That I’m Having to Navigate”: Reflections on Qualitative Approaches From Behavior Analyst Researchers

**DOI:** 10.1007/s40617-025-01097-2

**Published:** 2025-10-01

**Authors:** Victoria Burney, Ebonee Hodder, Sehar Moughal, Samantha van der Werff, Stephanie Xie

**Affiliations:** School of Psychology, The University of Auckland, Private Bag 92019, Auckland, 1142 New Zealand

## Abstract

Researching qualitatively can act as a catalyst to critically evaluate assumptions of objectivity and reflexivity. This article provides reflections of researchers, trained (and practicing) in behavior analysis, who are navigating diverse qualitative research in quantitative spaces. Influenced by critical collaborative autoethnography, this article provides our individual reflections processed collectively during group conversations. Through these discussions we reflect on the tensions we have experienced as behavior analysts engaging in qualitative research. We explore and unpack what qualitative approaches have prompted us to question: what is science, where is truth located, who has knowledge, and how is it accessed? This article may look different to the research behavior analysts typically read; we hope this stimulates an imagination for all that qualitative research can offer, while simultaneously avoiding an uncritical idealization of these approaches. We aim to model discomfort within the article, as a powerful tool to think reflexively about how these tensions align with our own values as researchers, and those of the field at large.

## Scene

[INT. SPACIOUS ROOM WITHIN UNIVERSITY GROUNDS, FLORECENT LIGHT. MIDDAY. THE SUN SHINES OUTSIDE.]

*Narrator: Sitting around the mahogany table are five discussants, deep in conversation about their experiences in qualitative research. An audio recorder sits in the middle of the table. Stephanie reaches out to switch on the recorder. Suddenly, all conversations stop. There is an uncomfortable silence. All five gaze at the audio recorder. They laugh. They are being recorded. They feel vulnerable, even as they control the data and resulting narrative. Introducing themselves to the audience seems a natural step to ease the tension.*

## Introductions

We are a group of researchers, colleagues and friends, who studied behavior analysis at different times through the same university verified course sequence. Four of us are Board Certified Behavior Analysts (BCBAs) working in clinical practice alongside undertaking further research. We are all currently engaged in doctoral-level study in various fields (such as education, critical psychology, behavior analysis) utilizing a variety of qualitative methods. As part of our doctoral studies, we are researching with diverse populations: South Asian women who have experienced family violence, parents engaged in behavior interventions for their children, care-experienced young people, neurodivergent school non-attenders, and communities focused on waste minimization, and we are researching across diverse systems: the state care, education, health, and justice systems. Our methodologies encompass intersectional feminism, critical autoethnography grounded theory, interpretive description, action research, and participatory research, among others.

Sehar’s doctoral research focuses on a therapy framework for South Asian migrant women who have experienced family violence. Sehar is using a critical feminist auto-ethnographical methodology to de/construct and reimagine therapy for brown, desi (Venkatraman, 2021) women. Sehar is a 1.5 generation immigrant (Bartley & Spoonley, 2008) and considers herself a “betweener” in spaces (Diversi & Moreira, 2016) when re/searching with her migrant community. As part of embodying qualitative methodologies, Sehar has and is continuously unpacking her own privilege (as a professional and researcher) in how she speaks, writes, and does research with brown, desi women who have lived experiences.

Ebonee’s doctoral research is a participatory exploration of the educational experiences of young people in state care through art. Ebonee has been working with young people navigating the care system for several years. She researches as part of a research collective which has been created out of her doctoral project, in which she is the only non-care experienced researcher. Together this collective is critical of the state and centers the importance of lived experience as knowledge.

Victoria’s doctoral research explores experiences of parents and clinicians within behavior analytic interventions for children. Her research draws on interpretive description as an approach for making sense of the perspectives of those on both sides of behavior analytic interactions. As a behavior analyst who is not a parent, Victoria sits as an insider and outsider within the research (Dwyer & Buckle, 2009) and takes a primarily constructivist perspective in her research.

Samantha’s doctoral research aims to better understand the school-related factors (or barriers) that contribute to the school refusal behavior of neurodivergent adolescents from the perspectives of young people, their caregivers, and their school. Samantha is consistently reflecting on her position as a neurotypical clinician working and researching with neurodivergent young people in schools. She is in the early stages of her research and is still navigating the nuances and complexities of qualitative research methodologies.

Stephanie’s doctoral research is grounded in principles of community and behavioral psychology that work alongside two communities driven to enhance their environmental practices. As a climate activist, Stephanie is excited to be combining the use of action research and behavioral approaches to co-create change in an organization and a residential suburb. Stephanie began her journey with a positivist way of knowing, but with time, shifted to being more aligned with a constructivist approach where knowledge is co-produced and jointly created.

The Narrator is the guide who takes our audience through unfamiliar or novel terrain; the narrator clarifies concepts and ideas specific to qualitative research that may be less familiar to behavior analysts.

*Narrator: While the introductions above may seem unusual, positionality statements such as these are considered critical in qualitative research. One way to start thinking about the concept of positionality is to reflect on one’s historical and situational contexts and learning histories, and how that may influence what and how one engages in research. For example, Sehar’s reflections on her experiences of being a migrant young person and accessing therapy services shaped her current doctoral research. Conversely, Sam’s clinical work as a behavior analyst working in schools inspired her research, not her lived experience. These differences impact how research is designed, conducted and understood. Readers may notice that our discussants use tacts such as “betweener”, “outsider”, “constructivist” and “positivist” in these positionality narratives. They are signaling their research paradigm and epistemological perspectives (ways of knowing; *Tuli, 2010*) for the community of readers. *Table 1* offers a glossary of some of these tacts, specific to qualitative research. Further, the researcher’s note their methodological commitments within their research projects (including “interpretive description”, “critical feminist autoethnography” and “participatory”). These terms help to identify (and justify) for an audience familiar with qualitative approaches what methods, tools, techniques, and analytic strategies the researchers will use to conceptualize, develop and achieve a credible (and consistent) research output (*Braun & Clarke, 2023*).*

**Table 1:** Glossary of key terms used within the article

Term	Description	Further reading
Action research	A participatory process that weaves together theory and practice (social action) and which contributes practical solutions to issues of pressing concern to people.	Reason & Bradbury (2008)
Autoethnography	A methodology where self (auto) is both the participant and researcher, and personal experiences are generated and analyzed as data to interpret the social or cultural world one is situated in (ethnography).	Ellis et al. (2011)
Betweener	Describes the position of the researcher who is neither a complete member of (insider) nor fully separate from (outsider) the group, population, or community they research.	Diversi & Moreira (2016)
Collaborative autoethnography	A branch of autoethnography where two or more researchers (participants) collect and analyze their personal and collective experiences as data to interpret their shared worlds.	Chang et al. (2013)
Construction	The interpretive process through which researchers generate meaning from the data, grounded in an understanding that knowledge is co-produced and shaped by researcher’s lens, values/assumptions and engagement with the empirical material (data).	Lincoln & Guba (1985)
Constructivist perspective	A research paradigm (subset of the interpretive paradigm) that seeks to understand and describe human experience and meaning making; “truth/reality” is socially constructed and varies depending on who participates and their cultural, societal and historical context. Science is intertwined with values.	Mayan (2023)
Greening	The process of becoming more active in protecting the environment to address environmental concerns, like pollution, recycling or waste minimization.	Crane (2000)
Insider/outsider (researcher)	Describes the relative position of the researcher as a member of (insider) or separate from (outsider) the group, population, or community being studied or researched.	Asselin (2003)
Interpretive description	A methodological framework (way of organizing qualitative research) concerned with the subjective experiences of people with an aim to generate clinically relevant descriptions and explanations (often with a practical or ‘so what?’ intention).	Thorne (2008, 2018)
Methodological commitments	The specific choices and principles that impact how research is carried out in practice (e.g., choice of methods, analysis strategies), as based on researcher’s research paradigm.	Patel & Patel (2019)
Participatory approaches	An approach to research that is committed to meaningfully working with members of the community on all stages of research, from identification of research problem, designing the study, data collection, analysis to mobilize action through social action.	Mayan (2023)
Positivism	A research paradigm that sees there being a truth that is generalizable and universal; sees truth as measurable and verifiable via precise observations. This approach positions science as values free.	Park et al (2020)
Research paradigm	A philosophical foundation, or lens, through which researchers view the world (e.g., positivist, constructivist, interpretive). These impact how research questions are conceptualized and selected, and how findings are interpreted.	Omodan (2025)
Unpack privilege	A recognition and understanding that certain elements of our identity (race, gender, age, education) afford us more privileges than others.	McIntosh (1990)

## Contextualizing the Product

Early in our research journeys, all of us realized the need for qualitative approaches in our research. Adopting qualitative approaches versus embodying them in our research are not the same. We are learning that the latter takes time; unpacking our own positionality (Pritchett et al., 2022), moving away from the idea of an objective researcher with all the answers, or the right answers (Ruiz & Roach, 2007), honoring different knowledges (Marecek, 2003), and embracing humility and discomfort (Dettmering & Hodzic, 2024).

Our writing and reflections are influenced by critical collaborative autoethnography, a process that empowered us to engage in a collective examination of our individual experiences, to understand the broader collective experience of using qualitative methodologies in our doctoral journeys (Chang et al., 2013), especially when working with and across diverse and marginalized communities. The added critical layer allowed us to examine “the boundaries of power relations between the researcher and the researched for the specific purpose of bringing about social change” (Bhattacharya, 2008, p. 306). As behavior analysts, this autoethnographic approach afforded us space to critique the values and approaches of the culture of behavior analysis from within the culture, as insiders who share these cultural links and assumptions. As a result of this process, the outcomes (this writing) are based in, but extrapolated from, the original data products (group discussion and interviewing). We present our writing as a critical conversation between peers focused on the potential for, and challenges with, embracing qualitative methods in our field. Our writing embodies the amalgamation of our individual and collective qualitative journeys to serve as a model, albeit messy. Generating this product involved its own tensions and challenges, which we have represented in the writing. We present our reflections in this way to normalize (and expose audiences to) an approach to presenting qualitative work which differs markedly from a typical dissemination of behavior analytic research.

*Narrator: Although this article embodies elements of collaborative autoethnographic approaches, it is just one example. Readers who are curious about ethnographic approaches more generally could explore recommended readings offered in *Table 1*.*

## Tacting our Function

Despite being at different stages of our doctoral research, we come together in this project to share our experiences of conducting qualitative research grounded in behavior analytic perspectives (informed by our behavior analytic training, certification, and practice), and aligned with the field of behavior analysis. In doing so, we reflect on this process and make sense of the tensions we have faced as we continue to grow and develop our skills in using qualitative methodologies.

In light of recent calls to encourage behavior analysts to be curious about qualitative investigation and what it can offer us as an approach (Burney et al., 2023), we aim to prompt readers to attend to and reflect on their own (research) practices, challenging their assumptions of what constitutes proper and scientific research. This reflection is necessary before delving into qualitative methodologies, to increase the likelihood of effective practice and decrease the likelihood of causing harm while navigating these relatively novel spaces.

*Narrator: The recording continues; the discussants are now participants. They think they understand what it feels like for research participants to be recorded, measured, and analyzed. Yet, they are privileged. They shape the narrative. Qualitative methodologies have exemplified this knowledge for them all, of their privilege. Yet they are still students. They ponder “how will we be seen by our behavior analytic community? Are we doing this the right way?”*

## Tensions we are Navigating

### Researching Objectively: “It’s Difficult to Argue that Data Ever Truly Speaks for Itself”

Our conversation begins by exploring what behavior analysis means to us, and how the science of behaviorism can diverge from, or sit uncomfortably with, qualitative approaches. We explore the idea that qualitative research centers people’s experiences, thoughts, and views (Braun & Clarke, 2019). We discuss and reflect on how different this is from our training in the field, where we were taught that as behavior analysts we hold the knowledge and expertise to draw out the objective “truth” in a situation (see Bowman et al., 2024, for exploration of this belief).

For all of us, applying a qualitative lens to our investigations involves a tension between the conceptual and methodological commitments of qualitative methodologies, and the long held, embedded commitments of behavior analysis as an objective or hard science (Johnston et al., 2020). When applying qualitative approaches in research, we discuss whether the behavior analytic commitment to being objective, or letting the data speak for itself (Dittrich, 2020; Gioia, 2021), is incongruent with post-positivist approaches to knowing (Braun & Clarke, 2023) which accept there is no answer (or truth) out there for us to fully uncover (or construct) without bias. If subjectivity is inherent in qualitative ways of researching (Flick, 2022), can this research be considered behavior analytic?

**Victoria**: I feel like my learning history as a behavior analyst was that I was an objective scientist: observing, recording, measuring, implementing. That I was almost invisible in my research. It didn’t matter who was researching, they would come to the same result... because it’s objective.

**Ebonee**: We were taught to look at data through a positivist lens to achieve a goal. That the data speaks for itself. You either got 80% correct across two sessions or you didn’t. But I think in a qualitative space it’s difficult to argue the data ever truly speaks for itself, you are interpreting that conversation based on your own meaning of what that person is saying. This acts as a stepping stone to think, well then maybe no data ever truly speaks for itself.

**Sehar**: I struggled with being objective when re/searching with women in my community. And I saw my struggle as a personal failure. I was not a very good scientist. Delving into the qualitative space allowed me to reflect on and accept different ways of knowing, being, and doing.

**Victoria**: We strive for objectivity, that’s the behavior analytic imperative, right? The data is IT. But in my research, this positivist positionality and reaching for objectivity doesn’t work. Because I am taking parent stories, and interpreting these through my lens, my learning history, my own experiences. What I know and feel and understand shapes the data, and if it’s not objective, is it any good?

*Narrator: When the group refers to positivism, they describe a paradigm which assumes there is one reality that can be identified, measured, and explained (*Lincoln et al., 2011*). Critical to positivism is the epistemology that researchers must be objective and separate themselves from their participants. In a positivist framework, this objectivity is the cornerstone of good research, and it is with this good research that the “truth” can be found (*Park et al. 2020*). Our discussants are grappling with discomfort that arises in balancing a commitment to objective science (and a positivist framing in behavior analytic research and practice) when faced with diverging needs and perspectives of their research communities (i.e., what is socially valid and important for participants to discover through research). They wonder how other behavior analysts who are using qualitative approaches which depart from a positivist lens reconcile such a disparity?*

*Narrator: The discussants all pause. Deconstructing the notion of objectivity is difficult for them. The air is thick with tension. They are passionate about the science of human behavior. But what does “science” mean in behavior analysis? And how critical is the commitment to objectivity in behavior analytic research?*

The discussion of objectivity stimulates further conversation about the inherent risk involved in qualitative research, and the need to proceed with care. Specifically, qualitative research (particularly drawing on social justice or critical, feminist positioning) has a responsibility to represent the voices and participants in ways that promote equity, justice, and highlight the concerns and issues most salient to participants themselves (Pritchett et al., 2022). We discuss this tension in line with researcher accountability to those who contribute to, or shape, the research: the participants. Specifically, we each describe the weight of responsibility we feel in representing our participant groups in ways that they would be comfortable with. In her participatory research, Ebonee reflects “when you are writing about people, and those people are your co-researchers and you have to, like, look them in the eye and say, ‘I wrote this about you, about us.’ it really makes you think.” Stephanie shares similar experiences as she interviewed members of the community as part of her doctoral research, particularly in interpreting statements from mana whenua (Indigenous people of Aotearoa), where she felt an acute sense of responsibility in wanting to ensure that her interpretations of their voices were accurate, and didn’t risk oversimplifying the depth of the metaphors shared.

The conversation shifts; given a move from valuing objectivity toward embracing subjectivity, discussion centers on meeting our ethical responsibilities in qualitative approaches. Each of us went through a rigorous ethics application process before gaining approval for our research projects, including some consultation with our research communities. As behavior analysts, we also adhere to our BACB Code of Ethics (Behavior Analyst Certification Board, 2020) in practice and in research, particularly in relation to mitigating any potential harm that may come from holding multiple relationships. Is it a conflict of interest to research with communities where we have the potential to hold dual roles? Or does this potential duality instead create opportunity for robust investigations to occur which prioritize participant voice and evoke agency?

Sehar has a strong sense of belonging to her community and has found recruiting participants to be faster than anticipated, even with strict inclusion criteria to ensure safety and protection of all participants. Sehar has spent a total of approximately 100 hours with her participants over six months with zero attrition. Given the vulnerability of her participants (with lived experiences of family violence), Sehar’s familiarity as a brown woman has been key to the ongoing research alliance. Wearing her betweener shoes, Sehar continuously reflects about the complex contexts of her participants, her own privilege as a researcher and how brown women are portrayed in objective research/writings. For Victoria, the shift from working clinically with parents in behavior analytic interventions, to interviewing with parents for the purposes of research, raised questions around the ethical imperative to avoid conflicts of interest during recruitment. Victoria questions if she would have different insight had she researched with parents that she had worked with before, where levels of familiarity and trust were high. She experienced discomfort in making ethically informed decisions to exclude existing (or past) clients from her research, where including these individuals might have bolstered the outcomes of the research, in ways that better represent the group Victoria hoped to serve with the project. The community within which Ebonee works (adolescents in state care), is incredibly small, comprising roughly 3000 youth across the country and as such, she knew it was likely that care-experienced young people she had worked with directly or indirectly would contact her to engage in the study (despite Ebonee not recruiting them directly). She had believed going into her doctoral research, that these relationships prohibited participant safety and power, but on reflection Ebonee felt it unfair to exclude people who were interested in engaging in the study because she had known them. These existing relationships, she found, fostered discussions that went deeper and further than participants who were strangers to her. Ebonee reflects on the ways that these relationships allowed participants to feel safe and autonomous; to know the type of person that was conducting the research prior to agreeing to participate and trusting she would act safely with their words.

*Narrator: Particularly in projects which embrace participatory research, feminist research, or research with historically marginalized communities, situations where the researcher and participating community have existing relationships can sometimes be protective, allowing for issues of power imbalance to be addressed in ways which prioritize the social validity and wellbeing, and impact of the research for the communities it serves (*Duea et al., 2022*, *Iqbal et al., 2023*).*

*Narrator: Stephanie takes a sip of water. Samantha stretches at the table. The tension in the conversation shifts, and the reflections continue around the engagement with research communities as part of qualitative research.*

### Reflexive Spaces in Objective Places: “That’s Just Like, the Other Stuff I do in the Car.”

We examine the tension of navigating reflexive practices within spaces striving for objectivity. We collectively see reflexivity as one of the greatest models given to us by qualitative methodologies. We reflect on how qualitative methodologies have acted as springboards in our own practice to be reflexive and think meaningfully about questions we feel often go unasked, particularly within behavior analysis. These are questions about power and who has the right to research, about data and through what lens we see it, and about social justice and whose aims we give legitimacy. Despite the reflexive tools qualitative research has given us, we collectively navigate the trap of overly romanticizing qualitative research or viewing it as inherently a more just, or valued, method of knowledge production.

For many of us, the step into qualitative research was the first time that we had meaningfully encountered the idea of reflexivity. We reflect on how this idea felt incongruent with the ideals of objectivity in science, which we had been taught to strive for.

**Sehar:** So, the assumption is that as soon as you come into this research space, you remove all your feelings and emotions and all your biases, and you’re an objective researcher, right? Which is bullshit.

**Samantha:** Yeah, I definitely wasn’t as intentional about reflecting on this in my clinical practice, anywhere near as much as I have been in planning my research. I didn’t even know what many of these words meant, until I was doing my doctoral research, honestly.

**Victoria:** Yeah... I bracketed off all those reflections as non-behavioral. I tried to put all that away. In my head, reflecting on my sessions, my interviews—whether they went the way I wanted, the way they needed to unfold—that was not my behavioral work. That’s just, like, the other stuff I do in the car.

**Ebonee**: For me it was almost the opposite, in my clinical space, we do a lot of work around the young person’s voice being in the center and so much of what we do is unpacking our own positionality and privilege. So, it was strange that when I initially came into the research space, I was thinking that I already knew what was needed, when in my clinical space, I really don’t work like that.

**Sehar:** It was more of a visceral experience for me. I did my master’s research in ABA with South Asian women who had experienced family violence, but I only included objective data. I did not give merit to processes, findings, and reflections that were other and not ours or scientifically based. But for my current doctoral project, I was able to pause and reflect on my previous research experience and my situatedness (lived experiences). Through embracing different ways of doing research and critiquing dominant narrative about brown women in therapy, I am giving myself the permission to have closure from previous tensions I had felt but could not name.

*Narrator: Reflexivity requires researchers to critically examine how their learning histories, context, position in the world, and beliefs, impact the research in iterative and cyclical ways (*Lazard & McAvoy, 2020*). Reflexivity is often seen as the core difference between quantitative and qualitative research. While there has been a recent push for quantitative researchers to engage in reflexive practice (*Jamieson et al., 2023*), it has long been a celebrated, even required practice when researching qualitatively (*Wren, 2004*)*.

*Narrator: As the conversation touches on ideas of reflexivity and humility, the group becomes more animated. They speak louder, people talk over each other, and heads nod emphatically.*

Stephanie expands the discussion of reflexivity, drawing on the behaviorally based food scrap trial she supported food businesses with. At the time of the year long trial, the service was free and fully funded by local government. This element of the trial being free of charge was a selling point to get businesses to participate, given that many openly shared their financial struggles with Stephanie. A year on, following business’ successful adoption of the food scrap segregation process, funders indicated that a continuation of services would only be feasible if the costs were passed onto businesses. This was to Stephanie’s horror (and disappointment), to realize that while her research yielded wins for her as a doctoral candidate, wins for the environment via streamlined waste segregation practices, and yet, acted as an additional stressor or burden for the involved food businesses. Stephanie considers how different the “intervention” could be, if food businesses were provided the opportunity and were consulted on the choice of goals early on. Perhaps, a completely different, yet more contextually appropriate approach to greening their workplace. Such reflections come with humility (Kirby et al., 2022), and recognition that in some instances a behavioral intervention with the best intention may result in unintended, sometimes even undesirable, consequences for participants.

As reflexivity requires us to continually analyze and critique ourselves in the research process, it can result in feelings of discomfort (Cumming‐Potvin, 2013). Reflexivity is a core tenant for critical thought. It allows us, importantly, to be critical of ourselves and our own research, which can lead to feelings of uncertainty. Victoria experienced this increased focus on reflection as both a gift and a challenge resulting from her experiences designing and doing qualitative research. In one sense, reflecting deeply on the process and products of qualitative data collection via interviews prompted generalization, and Victoria found herself being more reflective in clinical practice. Equally, Victoria found that this focus on reflexivity at times paralyzed her skills in problem solving and decision making when on the ground with parents in intervention spaces; contributing to a deep sense of imposter syndrome (see Apgar & Zerrusen, 2024, for a definition). As the conversation continues, Samantha and Stephanie share their feelings of vulnerability. Samantha reflects on being the least experienced in the group; Stephanie speaks of not having the host of clinical experiences others have. These perspectives are met with encouraging words and reassurance; that diverse experiences and perspectives are what makes this an interesting melting pot. Samantha further reflects that she is grateful to have a safe space to explore something new and shares her excitement around the opportunities and possibilities of qualitative research.

*Narrator: Everyone smiles, the room feels warm. A stream of afternoon light passes through colored glass panes, casting shadows on the carpeted floor. The discussants continue their conversation.*

While stepping into qualitative research gives us excitement for reflexivity and critical engagement, we are careful to avoid an uncritical stance that qualitative methods are inherently superior. There remains much qualitative research that lacks critical and reflexive analysis (Braun & Clarke, 2023). Wren describes that “rarely now, do qualitative researchers go beyond orthodox assurances about reliability and validity (largely a fairly narrow concern with method) to a more critical exploration of their constructions of the empirical material (a concern with how knowledge is generated).” (2004, p. 176). During our conversations, we pull each other back from getting caried away in our excitement of qualitative methods. Particularly, when claiming that qualitative research is itself a solution to the problems of power, and knowledge legitimacy, which we are critiquing. Samantha and Ebonee discuss the idea of exploitation within research, and how quantitative and qualitative research can both exploit and harm participants. Samantha reflects on the ways that PhD research, quantitative and qualitative alike, may often sit on a proverbial shelf and be read by no one, which may benefit the researcher at the expense of the community. However, she wonders about the different types of responsibility felt when hearing people’s stories and thinks about the ways that qualitative methodologies encourage us to disseminate reflexively in light of this responsibility.

Through reflexivity, qualitative research allows us to understand “dissemination as a reflexive and on-going conversation that one has with others as much as oneself “(Barnes et al., 2003, p. 161). Ebonee considers the way that qualitative research can create harm to participants through damage-centered narratives, where researchers portray communities as broken and powerless (see Tuck, 2009 for further discussion). She thinks of the ways the care-experienced community are often portrayed in these ways; written *about* as damaged instead of *with* as complex and resourceful. She reflects that one solution to these damage-centered narratives is community led, relational research between academic and community co-researchers, where co-researchers have decision making power in deciding the research tools (qualitative or quantitative) to achieve the research goals of the community.

Collectively, we reflect on arriving in the qualitative research space with a shallow idea of expertise. For many of us, we were navigating tensions that the qualitative research was simply to inform the “real research”: the behavioral intervention. We saw these interventions as the need and saw ourselves as the experts in their provision. We all recount experiences of needing to reframe qualitative approaches from *informing* the real work of intervention studies, to position qualitative approaches at the center of the investigation *as* the real work, standing on their own. We all entered into our doctoral projects assuming there would be a quantitative small-n component to this work, largely because that is what is selected for and preferred in our field. Some of us, like Stephanie, retained this intervention element. In her work to support the “greening” of the hospital, qualitative components of the project revealed insights from staff that accurate waste segregation has been a consistent challenge within their department, and a small-n behavioral intervention then grew out of this. For others, there has been a coming to terms with the value of qualitative research in its own right. Victoria entered her project with the view that qualitative data would inform a small-n pilot study around engaging parents, to ensure the research outputs were legitimate and valued by the field of behavior analysis. This perspective was challenged when including parent voices in the project. Victoria experienced a shift to acceptance that research from a qualitative lens is equally valuable and impactful, providing a way to elevate parent experiences within academic outputs. Ebonee reflects on her value that research should end in some sort of action, and sitting with the possibilities of what action qualitative research can achieve on its own. Sehar has finished piloting her therapy framework with a small number of South Asian women who have experienced family violence. When Sehar is asked about the graphs, Sehar is silent; there are none. Yet, the stories from her field work are rich and critical. No, it is not an intervention. Instead, Sehar’s use of qualitative methodologies has allowed her to shift responsibility away from her participants to researchers and professionals.

*Narrator: A phone alarm goes off. The conversation comes to an end. Stephanie switches off the recorder and the discussants sit in silence for a moment. There is a collective sigh of relief and a sense of fatigue.*

[INT. LATE AFTERNOON. RAIN ON THE WINDOWS OF THE NOW DARKENING ROOM. FLORESCENT LIGHTS ARE TURNED OFF AND THE SPACE IS QUIET.]

## Closing Comments and Directions

This critical conversation is the product of ongoing reflection and sense making between five colleagues trained in behavior analysis (four of whom are practicing behavior analysts) who are researching in qualitative spaces, while drawing on behavior analytic conceptualizations. This work highlights the tensions that we have experienced, and continue to navigate, at the intersection of behavior analysis and qualitative approaches. By creating and presenting this work through an example of a collaborative autoethnographic approach, we aim to provide a model for how qualitative approaches can look, as distinct from reports of quantitative research familiar to behavior analysts. Far from providing instruction to other behavior analysts, or presenting verifiable or replicable information, we hope that this article and the reflections contained within will stimulate others to imagine the possibilities of qualitative research in behavioral spaces, despite apparent challenges. By tacting our discomfort as early career qualitative researchers, we hope to motivate others to engage in reflexive processes, even if - and especially when - this feels uncomfortable.

We encourage other behavior analysts who may be curious about qualitative methodologies to think critically about how these ideas align with their values and the imperatives of the wider behavior analytic community. Importantly, our suggestion that qualitative approaches can bear fruit in behavior analysis is not without caveats. Our collective experiences suggest that in much the same way as other approaches to research, poor quality or uncritically adopted qualitative methods will not progress our science, or field, and may instead contribute to additional harms and challenges. Explicitly, we advocate for considered, informed, and responsive incorporation of qualitative approaches within topics and spaces important to the communities we support, rather than piecemeal or hastily adopted qualitative research projects. Qualitative research requires more than a task analysis; it should, if done well, compel researchers to reflect and challenge assumptions about their own knowledge, positionality, and truth seeking. We aim to motivate readers to explore the importance of these methods for their own work, hoping these tensions can—as they have for us—cultivate growth for researchers and practitioners alike, as well as the field of behavior analysis.

## Data Availability

Data sharing is not applicable to this article, and no dataset for this manuscript is publicly available.
